# Annotations

**Published:** 1890-06-14

**Authors:** 


### Annotations.

The British Medical Benevolent Fund.

Doctors are quiet sort of folk. They neither proclaim their merits nor parade their distresses.. Yet their merits are not inconspicuous, and their distresses are neither few nor small. When a pension of £20 or £25 a-year is looked upon as temporal salvation by a man who has received a liberal education, and has failed to acquire a competency through no particular fault of his own, that man must be admitted to be in a state of very considerable mental and material distress. The British Medical Association has rendered considerable services to the medical profession since the date of its inception until now. Not the least of those has been the founding of a benevolent fund, and the entirely gratuitous administration of it. That fund, from small beginnings, has grown to generous dimensions, so that last year, according to the report just issued, so much as £7,000 was invested on its behalf. The fund has now an annual income of about £2,000, more or less; and it distributes this in grants, loans, and annuities of varying amount, though in no case exceeding, perhaps, £25 a-year. The class of persons benefited are aged and helpless medical men, or widows who have been left penniless. Those chiefly, but not entirely; for many doctors who break down from the stress and strain of life in middle age are almost more in need of pity and succour than even the aged. Their young children are still about them, and must be fed and clothed. The benevolent fund promptly offers its aid to such as these, and by a timely loan or grant enables them to pay the *locum tenens* to secure the imperative rest, and to keep the wolf from the door until health is restored and the sun of prosperity begins to shine again. The orphans of medical men who are left to the tender mercies of a world that is too busy to heed them have a sure friend in the benevolent fund. True, it cannot help so much as it would; but what it can do it does promptly and generously, and without the painful and often unnecessary humiliations to which so many poor beneficiaries are subjected. All this work, as we have said, is undertaken and carried out entirely without cost. Mr. Edward East, himself a busy medical man, voluntarily conducts the enormous correspondence of the fund; and also receives and distributes large quantities of clothing, which are sent in by many kind friends, and are most gratefully received by the poorer pensioners. We do not know any benevolent association which better deserves the generous help of medical men and the outside public. Dr. Sidney Phillips, of 21, Upper Berkeley Street, is the honorary financial secretary; and we understand that three or four times the amount of the present annual income are needed to keep pace with the growing distresses of the aged and helpless portion of the medical profession.

Board and Residence for a Lady.

"A most comfortable home is offered to a lady who will give slight assistance, and pay a small remuneration weekly." Casually glancing at one of the advertisement columns of the "largest circulation in the world," the writer was attracted by the peculiar meanness of the advertisement just quoted. A "lady" is wanted, and a "home" is offered. In the advertisement the "home" is set forth with all the majesty of capital letters; the "lady" is printed in the same type as the remainder. The feeling excited in the mind is one of intense pity for those ladies to whom such an advertisement may be supposed to be addressed. The curious inquirer wonders how anybody could have conceived such a method of securing help in the household as is here indicated, and of making the helper pay for permission to work. But the idea being conceived, one wonders where the courage, or shall we say impudence? came from which inspired the advertiser who made the idea public in the columns of a newspaper. The "lady" is required to give some slight assistance. What is the nature of the assistance? The mind ranges over a wide region of possibilities in the attempt to answer this question. There is, perhaps, a baby to be nursed; possibly there may be twins. Or the mother may be an interesting invalid, longing for a sympathetic companion, who will curl her hair, wash up the tea dishes, and give her six children their baths before going to bed. But speculation is entirely futile, because not the least hint is given as to what the nature of the "slight assistance" may be, or as to the meaning put upon the word "slight" by the advertiser. It is plain, however, that some sort of useful service is required of the "lady" wanted, and that the advertiser is determined not only to get the service for nothing, but also to make the "lady" pay for her board and lodging. We earnestly hope that no educated woman will be found to answer such an advertisement. Every woman owes something, first to herself, and then to her fellow women. However sorely straightened any lady may be, she had better even want than lose her self-respect. The humblest occupation that is recognised as work, and paid for as such, is to be preferred to such humiliating and beggarly dependence as is asked of the "lady" by this mean advertiser. Poor ladies should make up their minds to stand together firmly. If they would but be steadfastly loyal to each other they would soon cease to be insulted by shameless and impudent advertisements.

A Girl of Quality.

Never perhaps was sterling merit so much regarded as now, if it happens to be a kind of merit that the public can understand, and if also it happens to be able to get itself well into notice. We add these qualifying conditions for the comfort of those tens of thousands of obscure persons who are endowed with rare merits, but merits of a kind that the public does not at the moment value or that it knows nothing at all about. Miss Fawcett, who is the heroine of the hour, not only in academic, but in all generous circles, has the good fortune to be endowed with rare intellectual qualities of a kind that are generally appreciated, and also to be placed on a familiar eminence to which all men are periodically accustomed to look. For the moment she cannot but be supremely happy: the proud daughter of a prouder mother. Intellectual capacity coupled with steadfastness of purpose is not very common even among men; among women it is possibly rarer still. The male creature, who both admires and sympathises, wonders what kind of future is open to the girl of rare quality who is "above the senior wrangler." Nobody but perhaps an undergraduate looks upon the senior wrangler as a great man. He has in him the stuff that goes to the making of great men; but he is only as yet a glorified schoolboy, ready to begin, under the greatest possible advantages, the real campaign of life. Sometimes, unfortunately, he never wages a true life warfare at all. His college efforts and his college successes seem either to have exhausted his energies or to have been enough for his ambition. What will Miss Fawcett do? She is said to be "sweet" as well as mathematical. For her own sake we hope she is. The mere mathematician is an exceedingly peculiar creature. He lives in a world apart, and has the smallest measure of concern for the joys and the sorrows, the failures and the victories of his fellow mortals. That is endurable in a man, but would be intolerable in a woman. If Miss Fawcett is, as we hope, to have a happy and a prosperous life, we cannot but wish for her a large world of interests and pleasures other than those that are purely mathematical. Men do not envy women their intellectual successes; but they desire for them all the sweet and gentle qualities which make and ennoble the typical woman as well as the mere powers of reasoning and thought.

